# Disrupting the Link between Corporal Punishment Exposure and Adolescent Aggression: The Role of Teacher-Child Relationships

**DOI:** 10.1007/s10964-022-01666-6

**Published:** 2022-09-13

**Authors:** Aimee Neaverson, Aja Louise Murray, Denis Ribeaud, Manuel Eisner

**Affiliations:** 1 Department of Criminology, Anglia Ruskin University, Cambridge, UK; 2 Department of Psychology, University of Edinburgh, Edinburgh, UK; 3 Jacobs Center for Productive Youth Development, University of Zurich, Zurich, Switzerland; 4 Violence Research Centre, Institute of Criminology, University of Cambridge, Cambridge, UK

**Keywords:** Teacher-child relationship, Corporal punishment, Adolescent aggression, Longitudinal design, Developmental criminology, Risk and protective factors

## Abstract

Previous research has identified harsh parenting practices, such as corporal punishment, as a predictor of adolescent behaviour problems such as increased aggression. However, not all children who experience childhood corporal punishment develop increased aggression, making the illumination of factors moderating this link an important question for informing prevention. In the current study, an autoregressive cross-lagged panel model was used to examine teacher-child relationships as both a direct and interactive protective factor (via weakening the effects of corporal punishment exposure) in adolescent aggression. Data was used from the Zurich Project on the Social Development from Childhood to Adulthood (z-proso). Self-reported data was collected at three time points: age 11 (*n* = 1144, 49% female) age 13 (*n* = 1366, 49% female) and age 15 (*n* = 1447, 48% female). Results suggested having a positive teacher-child relationship was a direct protective factor against concurrent aggression. However, there was not consistent evidence for a moderating effect of teacher-child relationships. Implications of these findings are discussed.

## Introduction

Research on corporal punishment primarily focuses on the subsequent development of negative outcomes (Gershoff, 2002); however, recent research has begun to identify protective factors between corporal punishment and subsequent negative behaviour (Neaverson et al., 2020a, b). Corporal punishment is commonly defined as an action which causes pain, but not injury, while using physical force with the intention of correcting or controlling a child’s behaviour (Straus, 1994a). Corporal punishment can include actions such as slapping, spanking, pushing a child roughly, and hitting with an object such as a belt. Corporal punishment is differentiated from child abuse within previous research, where physical abuse intends to injure and corporal punishment does not (Al-Modallal et al., 2008). Previous research has linked childhood corporal punishment to various negative behavioural outcomes including depression and substance misuse (Burlaka et al., 2020), child anxiety (Liu & Wang, 2020) and increased aggression (Lansford et al., 2012). Corporal punishment is thought to lead to increased aggression as it can signal to children that aggression is the correct way to act as a means to reach their goals (Straus, 1996). Meanwhile, it fails to teach children alternative appropriate behaviours (Gershoff, 2013). However, not all young people who have been exposed to corporal punishment develop increased aggression. Understanding protective factors that help prevent the development of increased aggression amongst those exposed to corporal punishment can have implications for interventions to reduce the impact of exposure to corporal punishment. This is important because while there is a global movement towards outlawing corporal punishment, many youth are still exposed due to many countries still allowing corporal punishment and because it persists at some level even in countries where it has been made illegal. This study examines the direct and interactive protective effects of positive teacher-child relationships assessed at ages 10 to 15 between corporal punishment and aggression concurrently. Furthermore, this research examines the importance of a positive teacher-child relationship and addresses the gap in literature by examining teacher-child relationships as a protective factor in early adolescence.

### Corporal Punishment and Subsequent Adolescent Aggression

Parenting risk factors have been examined by previous research as an important predictor of childhood and adolescent aggression (Lansford et al., 2011; Eisner & Ribeaud, 2007). Previous research has identified several groups of parenting risk factors including, low parental involvement in children’s activities, poor supervision, inconsistent and harsh parental discipline and the lack of parental warmth or emotional support (Eisner & Malti, 2015; Loeber & Hay, 1997; Olson et al., 2011). However, evidence suggests that one of the most important child-rearing variables linked to aggressive behaviour is related to parents’ use of corporal punishment (Gershoff, 2002). Parenting practices that included punitive interactions such as yelling and using threats were also associated with disruptive behavioural problems such as aggression, as well as internalizing problems like depression (Stormshak et al., 2000). However, it was found that physically aggressive parenting specifically predicted child aggression.

It is important to apply a developmental framework when examining the impact of corporal punishment on a young person’s level of aggression. This is due to previous research finding that the strength of a risk factor can often depend on the stage of the young person’s development (Dubow et al., 2016; Fontaine et al., 2016). For example, during pre-school and adolescence, the family environment may have a greater influence when looking at the immediate impact of parental discipline practices. This is because during the pre-school and early adolescence age, the presence of the young person’s parents in their life is far greater. Furthermore, it is around age 11 that adolescents are learning to inhibit aggressive impulses as they develop increased cognitive control. A young person within this stage of early adolescence may be experiencing rapid hormonal change as well as sensation seeking, which could heighten sensitivity to social influences (Benson and Buehler, 2012). As the young person develops and enters the stage of later adolescence (age 15 + ), these hormonal changes may become more intense which could increase their levels of aggression as well as sensitivity to social influences and interactions with significant others within their daily lives, such as teachers.

One example of the mechanism by which corporal punishment can increase aggression is through the effect on emotional regulation. Emotional regulation is the process through which individuals’ control which emotions they have, when they have them and how they experience and express these emotions (Calvete & Orue, 2012; Gross, 2007). These emotions can be extrinsic when another person helps to regulate the person’s emotions, or intrinsic (automatic or effortful) when a person regulates his or her own emotions (Sheppes et al., 2014). Being unable to regulate one’s emotions has been found to be related to several forms of psychopathology (Aldao et al., 2010). With regards to emotional regulation being related to aggressive behaviour, previous studies have found that aggressive adolescents often use less effective emotional regulation methods than non-aggressive adolescents (Calvete & Orue, 2012; Nas et al., 2005).

It has been found that poor emotional regulation could explain how adverse parenting practices contribute to poor adolescent adjustment (Eisner & Malti, 2015; Morris et al., 2007). Harsh parenting practices are often associated with poor emotional regulation of the adolescent. Poor emotional regulation is then associated with aggressive behaviour from early childhood onwards. Children are more likely to develop aggressive behavioural patterns if they have shown deficits in affective regulation and impulse control (Krahé, 2001). These deficits make it more difficult to constrain their aggressive impulses and they are then often perceived as having a difficult temperament. The knock-on effect of this is that children can be treated differently by their social environment based on their temperament, including experiences at school.

Poor emotional regulation has also been found to be linked to physical aggression due to its effect on internal scripts and schema (Terzian et al., 2015). When children who have difficulty managing their emotions encounter a social situation that is emotion-arousing, they often rely on automatic scrips and schema rather than on unique cues (Terzian et al., 2015). They also tend to perceive fewer cues, generate fewer solutions and are more likely to select aggressive responses (Eisenberg et al., 2001; Terzian et al., 2015). Furthermore, adolescents with deficits in emotional regulation skills are more likely to display strong affect which can elevate risk for peer rejection and victimisation (Hubbard, 2001) and experience poor overall psychosocial adjustment (Terzian et al., 2015; Wyman et al., 2009).

Another possible causal link between corporal punishment and subsequent aggression is through social and cognitive skills (Eisner & Malti, 2015). For example, social disadvantage predicted harsh and inconsistent parenting, low supervision, and poor parent-child attachment (Dodge et al. 2015). This in turn, predicted social and cognitive deficits which predicted conduct problem behaviour. When children enter formal schooling with social and cognitive deficits, they are more likely to display conduct behavioural problems. High levels of conduct problems predict social and academic failure in elementary school, which in turn predicted parental withdrawal from supervision. Low parental supervision was associated with deviant peer associations which then predicted increased adolescent aggression (Dodge et al., 2015). Furthermore, if children lack social and cognitive skills and do not learn to regulate physical aggression during pre-school years, they are likely to develop increased levels of physical aggression later in life (Tremblay, 2004). This is due to the fact that if children see aggression as a legitimate form of social behaviour, they are more likely to demonstrate higher levels of physical aggression themselves (Erdley & Asher, 1998).

When considering the association between corporal punishment and aggression, there is evidence of differences for males and females (Gershoff, 2002). For example, there is a stronger association between corporal punishment and aggression for boys as boys tend to exhibit aggression more than girls and also may elicit more corporal punishment from parents than do girls (Gershoff, 2002). Furthermore, the frequency of spanking is higher for boys than for girls (Straus & Stewart, 1999) and girls are less likely to experience corporal punishment than boys (Taylor et al., 2011). Boys who experienced corporal punishment at age nine displayed increased levels of aggression during the following two years; however, there was no significant association for girls (Topçuoğlu et al., 2013).

### Positive Teacher-Child Relationship as a Protective Factor

Given that parents are often the primary attachment figure for children and adolescents, relationships with parents have been the main focus within attachment literature (De Laet et al., 2014). The parent-child relationship and its association with externalizing behaviours has been researched extensively (Doumen et al., 2008); however, research now also shows that the quality of a teacher-child relationship can shape the development of externalizing behaviour amongst young people (Silver et al., 2005; Talty et al., 2022). Despite the wealth of research and literature, teachers often underestimate the impact of a positive teacher-child relationship on healthy adolescent development (Davis & Dupper, 2004). Thus, the current study is important as it contributes to the understanding of the impact of positive teacher-child relationships on adolescent development and externalizing behaviours when exposed to known risk factors.

Various developmental theories have highlighted the importance of having a positive teacher-child relationship with regards to adolescent development, such as social-motivation theory, interpersonal theory, social bond theory and developmental systems theory (Sabol & Pianta, 2012). Each of these theories highlight the importance of emotional support for students as a means to foster healthy development for young people and support the argument that having a positive relationship with a teacher can play an important role in modifying classroom behaviour (Silver et al., 2005). Having a secure positive teacher-child relationship can function as a buffering factor as it can result in the development of positive affect and socially competent interactions with others (Hughes et al., 1999). It could also be suggested that having a positive teacher-child relationship could help prevent a young person from acting in an aggressive way, which, in turn, allows them to develop more prosocial behaviours. For example, having a positive teacher-child relationship can have an ameliorative effect on adolescent aggression (Blankemeyer et al., 2002). Aggressive young people who had a strong teacher-child relationship were found to be less aggressive in the following year. Furthermore, positive teacher-child relationships are important for the development of positive behavioural outcomes. Positive relationships with teachers protected against the risk associated with higher levels of disruptive behaviour in the classroom (Silver et al., 2005). Additionally, there is evidence of an association between positive teacher-child relationships and fewer antisocial behaviours (Tiet et al., 2010). Having a positive teacher-child relationship can act as a significant predictor of lower levels of antisocial behaviour for adolescents (Tiet et al., 2010). Furthermore, having a positive teacher-child relationship can have direct main effects on reduced antisocial behaviour and indirect effects on better youth adjustment (Tiet et al., 2010).

Having a positive relationship with a teacher has potential protective capabilities; however, the focus has been often on its “main” (sometimes termed “direct”) effect. It is also important to consider its interactive effects, i.e., whether having positive relationships with teachers can help break the links between experiencing harsh parenting at home and poor behavioural outcomes. In the criminological literature, distinctions between direct and interactive effects are considered important. A direct protective factor “predicts a low probability of offending” (Ttofi et al., 2016) while an interactive protective factor also predicts a low probability of problem behaviour, but it is considered a factor that moderates behaviour (Andershed et al., 2016; Lösel & Farrington, 2012; Ttofi et al., 2016). Positive relationships with teachers can act as interactive protective factors against future adverse behaviours (Stuhlman & Pianta, 2001). Students with mutually positive relationships with teachers reported fewer problem behaviours both concurrently and up to four years later (Obsuth et al., 2017). More recently, studies examined the protective effect of teacher-child relationships on young people’s delinquency using propensity-score matching and found that students who reported better relationships with teachers at age 10 also reported fewer delinquent acts at ages 13, 15 and 17 (Obsuth et al., 2021).

It is important that gender differences are considered when examining positive teacher-child relationships as an interactive protective factor. This is because the quality of a teacher-child relationship can differ for males and females (Blankemeyer et al., 2002). For example, teachers reported closer relationships and less conflicts with females when compared to males (Birch & Ladd, 1997). Furthermore, girls developed closer relationships with teachers when compared to boys (Choi & Dobbs-Oates, 2016). This is important to consider when examining the protective capabilities of a positive teacher-child relationship with regards to corporal punishment and adolescent aggression. Based on the above discussion, positive teacher-child relationships warrant further investigation as an interactive protective factor between corporal punishment and adolescent aggression.

## Current Study

While there is evidence that supports the link between teacher-child relationships and well-being in young children, less is known about positive teacher-child relationships as an interactive protective factor. No study to date has tested positive teacher-child relationships as an interactive protective factor between corporal punishment and aggression while accounting for previous levels of aggression. Thus, this study tested the hypothesis that positive teacher-child relationships moderate the association between corporal punishment and aggression in adolescence using an autoregressive cross-lagged panel model and longitudinal data. Based on the above-outlined considerations, it was hypothesised that children with stronger teacher-child relationships would be more protected against the adverse effects of corporal punishment, when considering adolescent aggression. This study also examined main effect and gender differences when considering the relation between corporal punishment, teacher-child relationships, and aggression thus making an important contribution to the field of criminology and adolescent development.

## Methods

### Participants

Data came from the Zurich Project on the Social Development from Childhood to Adulthood (z-proso). Z-proso is an experimental, prospective ongoing multi-rater longitudinal study of the development of aggressive and other antisocial behaviours based in a culturally diverse urban setting in Europe (e.g., Eisner et al., 2008; Ribeaud & Eisner, 2010, Ribeaud et al., 2022). The current analysis focused on the longitudinal component of the study.

The current study focuses on adolescence and thus uses the wave 4,5, and 6 data when participants were aged 11, 13, and 15; wave 4 [*N* = 1144, *M*_age_ = 11.3, 51% male (*n* = 583), 49% female (*n* = 561)]; wave 5 [*N* = 1366, *M*_age_ = 13.7, 51% males (*n* = 703), 49% females (*n* = 663)]; wave 6 [*N* = 1447], *M*_age_ = 15.4, 52% males (*n* = 750), 48% females (*n* = 697). In wave 5 (age 13) the initial eligible target sample (1675) could be re-contacted and actively consent to participate in the study. This resulted in an increase in the number of participants in later data collection waves (age 13 *n* = 1366; age 15 *n* = 14467), however, as only participants with data from all three waves were included in this study, it does not impact the outcomes of this study. When comparing the sample re-recruited at age 13 and the target sample, there were almost no differences in response rates by neighbourhood disadvantage or migration background (Eisner et al., 2019, Ribeaud et al., 2022).

### Procedure

In line with ethical requirements for conduct with human subjects in Switzerland, informed consent was obtained from parents at wave four (age 11) and from children at age 13 onwards (Ribeaud et al., 2022). Participants were administered pen-and-pencil questionnaires in the German language, the official language of Zurich. The questionnaires were completed in classrooms in 90-minute sessions in groups of 5–15 and guided by trained research students. Data included in the current study is all self-reported. Data was collected during school lessons at age 11, however at age 13 and 15 data was collected during leisure time. A cash incentive worth US$30 was given to participants at age 13 and at age 15 they received US$50.

Additional information of the study recruitment, attrition, measures, and sample characteristics can be found in prior publications (e.g., Eisner & Ribeaud, 2007; Ribeaud & Eisner, 2010, Eisner et al., 2019, Ribeaud et al., 2022) and on the study website: https://www.jacobscenter.uzh.ch/en/researchzproso/about us.html.

### Measures

To ensure methodological robustness, data from each measure used was required from all three waves in order to facilitate the analysis required to explore protective factors. Identical measures and variables were available for ages 11, 13 and 15 which made these waves of data methodologically suitable for this study.

### Gender

Child gender information was based on information collected from the first parent interview with males coded as 1 and females coded as 2.

### Corporal Punishment

Self-reported data on young people’s experience of corporal punishment was collected using parenting questionnaires that drew on the Alabama Parenting Questionnaire (Shelton et al., 1996) and the Parenting Scale from the Kriminologisches Forschungsinstitut Niedersachsen (KFN), adapted by the z-proso Project Team (Wetzels et al., 2001). Participants were asked to report their experience of three types of corporal punishment (spanking, slapping, pulling hair/ears) administered by their parents in the 12 months prior to the interview, on a 3-item scale from Never to Often (Cronbach’s α_age 11_ = 0.63; α_age 13_ = 0.70; α_age 15_ = 0.66). Scores for the three items were averaged to obtain a corporal punishment composite score.

### Aggression

Aggression was measured using the self-report version of the Social Behaviour Questionnaire (SBQ, Tremblay et al., 1991) adapted for adolescents (Murray et al., 2017). Self-reports were used because it is often the case that adolescents have less contact time with their parents due to the increased time spent with their peers and being out of the home, resulting in parents observing less of their behaviour (Marcus, 2017). The present study measured adolescent aggressing using a mean-score scale which included 9 items on aggressive behaviour; higher scores indicate greater aggressive behaviour (Cronbach’s α_age 11_ = 0.77; α_age 13_ = 0.84; α_age 15_ = 0.83). Three SBQ items each assess reactive (e.g., you reacted in an aggressive manner when teased), proactive (e.g., you scared other children to get what you want) and physical aggression (e.g., you physically attacked other people) in the last 12 months on a 5-point Likert scale from *never* to *very often*. It is important to note that physical aggression items do not specify whether the aggressive acts were proactive or reactive, whereas the proactive and reactive aggression items make more explicit reference to whether the aggression was instrumental or in response to provocation. However, previous research on this sample has supported the internal consistency and concurrent and factorial validity, as well as metric invariance across adolescence, sensitivity to intervention, and resistance to response shifts of SBQ scores (Murray et al., 2017; Murray et al., 2017; Murray et al., 2018, 2019; Murray et al., 2016, 2019; Murray et al., 2016).

### Teacher-Child Relationships

Positive teacher-child relationships were measured by asking students to report their relationship with their teacher. They did this by rating the following three statements on a 4-point Likert scale from completely untrue = “1” to completely true = “4”: “I get along with my teacher”, “the teacher is fair to me”, and “the teacher supports me” (α_age 11_ = 0.78, α_age 13_ = 0.77, α_age 15_ = 0.82). A mean score of their response was utilized to create the scale for the current analyses. If students had multiple teachers, they were asked to give an average response based on all of their current teachers. A self-reported measure of teacher-child relationships was used in this study as it was judged more important to measure how the young person perceived their relationship with the teacher, rather than how the teacher perceived it as the former is more likely to be a driver of a child’s behaviour.

### Analytical Procedure

Teacher-child relationship was tested as an interactive protective factor in the relation between corporal punishment and both concurrent and subsequent aggression using autoregressive cross-lagged panel models estimated in Mplus 8 (Muthen & Muthen, 1998–2017). Specifying an autoregression within the cross-lagged panel analysis allows the model to adjusted for past levels of aggression, experiences of corporal punishment and previous levels of teacher-child relationship. Product terms formed of centred predictors were added as this allows moderating effects to be tested. This method was used to test the hypothesis that having a positive teacher-child relationship could protect against the effects of corporal punishment on aggression both as a main effect and an interactive effect. Descriptive statistics and correlations were all run using IBM SPSS version 24.

Maximum likelihood estimation with robust standard errors (MLR) for parameter estimation was used to account for missing data and skewness. Comparative fit index (CFI), Tucker-Lewis index (TLI) and the root mean square error of approximation (RMSEA) were used to evaluate model fit. Although the chi-square is also reported for all models, it was not used in the evaluation of model fit due to the tendency of the chi-square to reject trivially mis-specified models for large samples (Hu & Bentler, 1999). Throughout, standardized regressions, coefficients or betas are presented and may be interpreted as indicators of relative effect size. To explore if there were differences in the protective capabilities of teacher-child relationships for males versus females, analyses were also conducted stratified by gender. Structural equation modelling is a confirmatory modelling approach that assesses the consistency of data with pre-specified hypotheses; however, it can be used in an exploratory fashion in the context of model generation (Jöreskog, 1993). In the current study, the possibility of introducing model modifications was allowed if initial hypotheses were not supported. In this respect, the analyses should be considered exploratory. Given the lack of previous studies examining the interactive effects of teacher relationships in the relation between corporal punishment and aggression in adolescence, this more exploratory approach was felt justified as it offered the production of new empirical findings of the protective effect of teacher-child relationships. Furthermore, as the method used employs an approach of exploring changes over time in the variables of interest using longitudinal data to infer the relations between variables, there is less need to adjust for potential confounds than in designs without repeat longitudinal measures.

## Results

### Descriptive Analyses

Mean levels of corporal punishment, teacher-child relationships, and aggression across the three timepoints is displayed in Table 1. Results show that mean levels of positive teacher-child relationships decreased as participants got older. With regards to gender differences, the results show that females had slightly higher levels positive teacher-child relationships when compared with males. This finding is consistent with previous research which found that teachers reported having stronger relationships with females than with males (Birch & Ladd, 1997).

**Table 1:** Descriptive statistics

	N	*M*	*SD*	Range	Skewness	Kurtosis
*Corporal Punishment*						
Age 11	1144	1.22	0.41	1–4	2.80	10.51
Age 13	1350	1.20	0.42	1–4	2.94	10.40
Age 15	1445	1.17	0.37	1–4	2.80	8.68
*Teacher-Child Relationships*						
Age 11	1134	3.47	0.59	1–4	−1.259	1.672
Males	576	3.39				
Females	558	3.56				
Age 13	1361	3.15	0.65	1–4	−0.710	0.066
Males	702	3.12				
Females	659	3.20				
Age 15	1446	3.06	0.67	1–4	−0.662	0.064
Males	749	3.03				
Females	697	3.10				
*Aggression*						
Age 11	1144	1.54	0.44	1–4	1.53	3.26
Males	581	1.65				
Females	563	1.43				
Age 13	1365	1.75	0.59	1–4.89	1.36	2.22
Males	703	1.90				
Females	662	1.59				
Age 15	1446	1.69	0.56	1–4.56	1.52	2.84
Males	749	1.81				
Females	697	1.56				

Correlations among the corporal punishment, teacher-child relationship and aggression variables are included in the Supplementary Materials (Table S1).

### Autoregressive Cross-Lagged Results: Positive Teacher-child Relationships

**Fig. 1:**
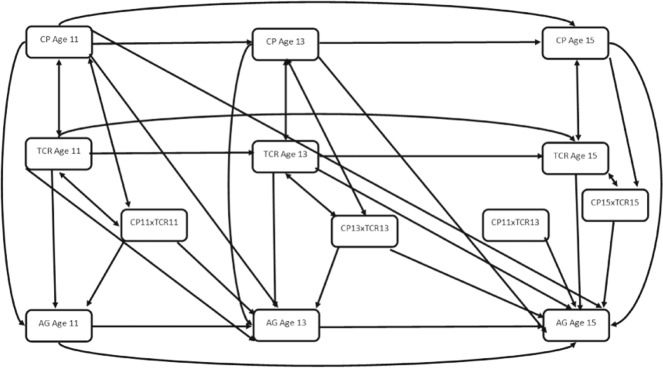
Autoregressive Cross-lagged Panel Model Testing Teacher-Child relations as a protective factor. AG Aggression, CP Corporal Punishment, TCR Teacher-Child Relationship. Lines with one arrow represent regression paths. Lines with two arrows represent correlational paths

The autoregressive panel model presented in Fig. 1 was fit to test positive teacher-child relationships as an interactive protective factor. All predictor variables (corporal punishment and teacher-child relationships) were first centred before being entered into the model. Interaction effects were tested by using product terms created by the centred variables. The initial model did not show a good fit to the data X^2^(36) = 162.38, *p* < 0.05, RMSEA = 0.06, 90% CI [0.05, 0.07], CFI = 0.83, TLI = 0.72. Modification indices were examined and recommended including the correlational path between product terms [age 11 corporal punishment × age 13 teacher-child relationship] and [age 11 corporal punishment × age 11 teacher-child relationship]. The inclusion of this correlational path resulted in a good fit to the data X^2^(31) = 64.73, *p* < 0.05, RMSEA = 0.03, 90% CI [0.02, 0.04], CFI = 0.97, TLI = 0.95.

**Fig. 2:**
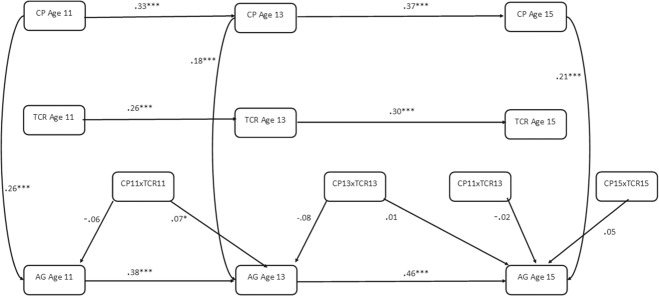
Autoregressive Cross-Lagged Panel Model Testing Positive Teacher-Child Relationships as an Interactive Protective Factor Between Corporal Punishment and Aggression. AG Aggression, CP Corporal Punishment, TCR Teacher-child Relationships. Lines represent regression paths. *p < 0.05, **p < 0.01, ***p < 0.001

**Fig. 3:**
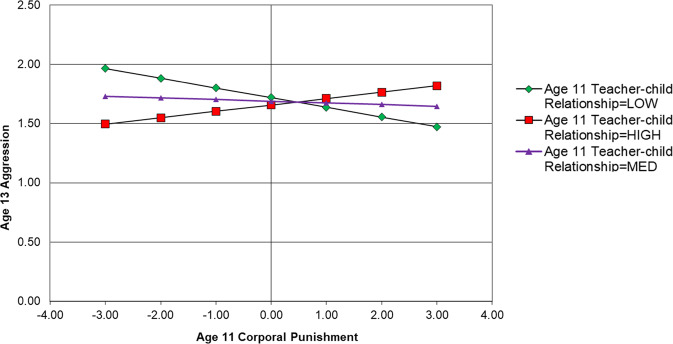
Simple Slopes Showing the Association between Age 11 Corporal Punishment and Age 13 Aggression at High (+1 *SD*) Medium (centred mean) and Low (−1 *SD*) Levels of Positive Teacher-Child Relationship for the Full Sample

Results of this model are displayed in Fig. 2. Only statistically significant paths relevant to moderation results are displayed, however, non-significant results not displayed are available upon request from the first author. The results show that having a positive teacher-child relationship had significant main effects against concurrent aggression (age 11, β = −0.277, *p* > 0.05; age 13, β = −0.211, *p* > 0.05; age 15, β = −0.138, *p* > 0.05) (Table 2). However, having a positive teacher-child relationship was not a significant interactive protective factor within the same timepoint at age 11 (β = −0.06, *p* > 0.05), age 13 (β = −0.08, *p* > 0.05) or age 15 (β = 0.05, *p* > 0.05). In terms of subsequent aggression, there was a significant interaction between teacher-child relationship at age 11 and corporal punishment at age 11 in predicting age 13 aggression (β = 0.07, *p* < 0.05). The direction of the interaction suggests that higher levels of positive teacher-child relationships exacerbated the effects of corporal punishment on subsequent aggression. As shown in Fig. 3, simple slopes suggest that those who reported higher levels of positive teacher-child relationships displayed higher levels of aggression after having been exposed to corporal punishment; however, this effect is modest in magnitude.

Results were not consistent when considering paths across different developmental stages in adolescence. For example, results show that having a positive teacher-child relationship at age 13 was not a significant interactive protective factor between age 13 corporal punishment and age 15 aggression (β = 0.01, *p* > 0.05). Having a more positive relationship with a teacher at age 13 was also not an interactive protective factor between age 11 corporal punishment and age 15 aggression (β = −0.02, *p* > 0.05).

**Table 2:** Path results of autoregressive CLPM testing teacher-child relationship as an interactive protective factor

Regression paths	Estimate	*S.E*.	*Est./S.E*.	*Sig*.
*Outcome Variable: Age 15 Aggression*				
Age 15 Teacher-Child Relationship (Centred)	−0.138	0.027	−5.192	<0.001
Age 15 Corporal Punishment (Centred)	0.206	0.041	5.053	<0.001
Age 15 Corporal Punishment × Age 15 Teacher-Child Relationship (Product Term)	0.054	0.036	1.499	0.134
Age 13 Corporal Punishment (Centred)	0.006	0.040	0.138	0.890
Age 13 Teacher-Child Relationship (Centred)	0.047	0.026	1.805	0.071
Age 13 Corporal Punishment × Age 13 Teacher-Child Relationship (Product Term)	0.003	0.050	0.061	0.951
Age 11 Corporal Punishment (Centred)	−0.001	0.036	−0.040	0.968
Age 11 Corporal Punishment × Age 13 Teacher-Child Relationship (Product Term)	−0.023	0.034	−0.692	0.489
*Outcome Variable: Age 13 Aggression*				
Age 13 Teacher-Child Relationship (Centred)	−0.211	0.030	−7.128	<0.001
Age 13 Corporal Punishment (Centred)	0.175	0.035	4.955	<0.001
Age 13 Corporal Punishment × Age 13 Teacher-Child Relationship (Product Term)	−0.080	0.044	−1.833	0.067
Age 11 Corporal Punishment (Centred)	−0.014	0.033	−0.409	0.683
Age 11 Teacher-Child Relationship (Centred)	−0.031	0.031	−0.988	0.323
Age 11 Corporal Punishment × Age 11 Teacher-Child Relationship (Product Term)	0.068	0.030	2.231	0.026
*Outcome Variable: Age 11 Aggression*				
Age 11 Teacher-Child Relationship (Centred)	−0.277	0.030	−9.110	<0.001
Age 11 Corporal Punishment (Centred)	0.256	0.040	6.427	<0.001
Age 11 Corporal Punishment × Age 11 Teacher-Child Relationship (Product Term)	−0.062	0.041	−1.513	0.130

*Est* parameter estimate, *S.E.* standard error

### Gender Differences

The main autoregressive cross-lagged panel model displayed in Fig. 1, with paths estimated freely for males and females, provided a poor fit to the data (X^2^(72) = 226.25, *p* < 0.05, RMSEA = 0.07, 90% CI [0.06, 0.07], CFI = 0.86, TLI = 0.76). The addition of the same correlational path recommended by the modification indices in the main model ([age 11 corporal punishment × age 13 teacher-child relationship] and [age 11 corporal punishment × age 11 teacher-child relationship]) resulted in a good fit to the data (X^2^(62) = 108.29, *p* < 0.05, RMSEA = 0.03, 90% CI [0.02, 0.04], CFI = 0.97, TLI = 0.93). The results of the autoregressive cross-lagged panel model for males and females is discussed separately in the following sections.

#### Males

**Fig. 4:**
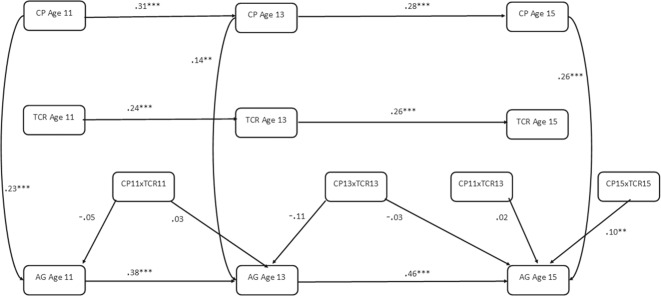
Autoregressive CLPM for Males when Testing Positive Teacher-Child Relationships as an Interactive Protective Factor. AG Aggression, CP Corporal Punishment, TCR Teacher-child Relationships, Lines represent regression paths. *p < 0.05, **p < 0.01, ***p < 0.001

**Fig. 5:**
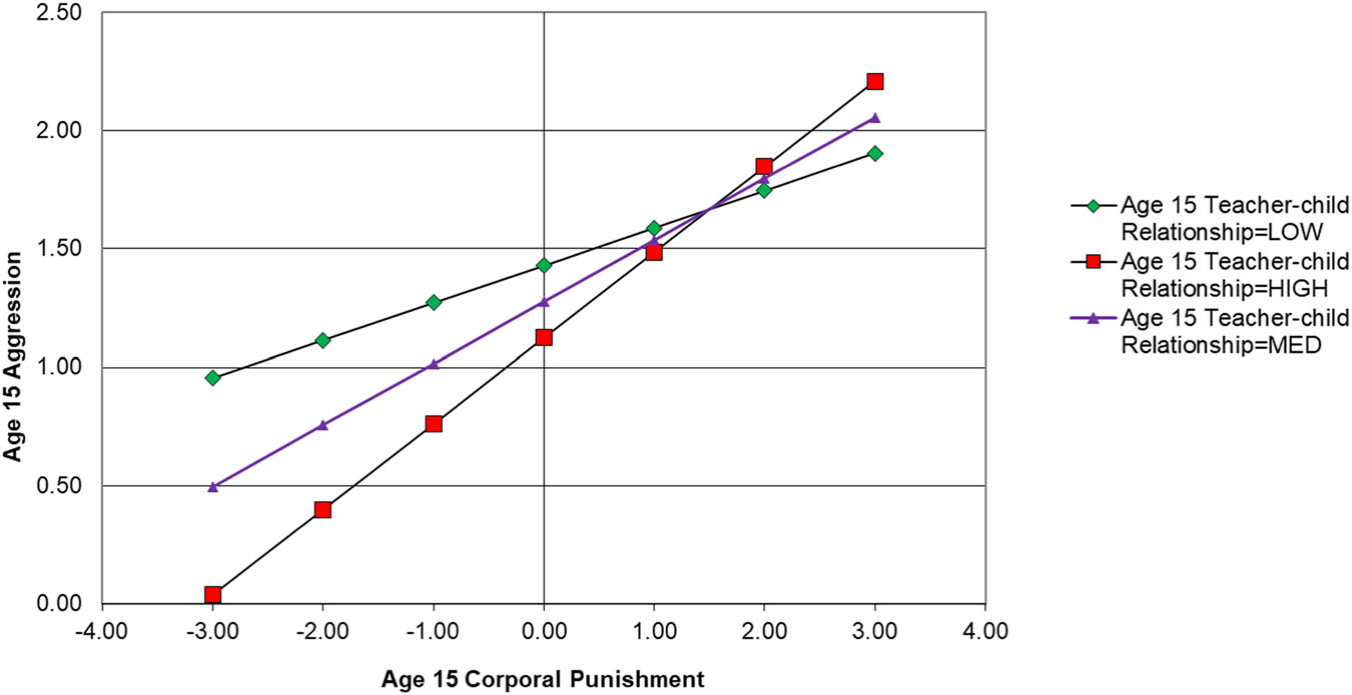
Simple Slopes Showing the Association between Age 15 Corporal Punishment and Age 15 Aggression at High (+1 *SD*) Medium (centred mean) and Low (–1 *SD*) Levels of Positive Teacher-Child Relationships for Males

Results indicated that there were significant main effects of positive teacher-child relationships on concurrent aggression for males (age 11, β = −0.300, *p* > 0.05; age 13, β = −0.205, *p* > 0.05; age 15, β = −0.153, *p* > 0.05); however, the strength of this effect varied by developmental stage and was stronger for earlier ages (Table 3). There was no significant moderating effect of teacher-child relationships at age 11 or 13; however, having a positive relationship with a teacher at age 15 significantly interacted with age 15 corporal punishment in predicting age 15 aggression (β = 0.10, *p* < 0.05; Fig. 4). The direction of the interaction suggested that having a positive relationship with a teacher at age 15 exacerbates the effects of corporal punishment on concurrent aggression. The simple slopes of this interaction are displayed in Fig. 5. In terms of lagged interactive effects, results indicate no significant moderating effects of teacher relationships on the relations between corporal punishment and later aggression.

**Table 3:** Path results for males of autoregressive CLPM testing teacher-child relationships as an interactive protective factor

Regression Paths	Estimate	*S.E*.	*Est./S.E*.	*Sig*.
*Outcome Variable: Age 15 Aggression*				
Age 15 Teacher-Child Relationship (Centred)	−0.153	0.036	−4.293	<0.001
Age 15 Corporal Punishment (Centred)	0.260	0.047	5.492	<0.001
Age 15 Corporal Punishment × Age 15 Teacher-Child Relationship (Product Term)	0.102	0.039	2.598	0.009
Age 13 Corporal Punishment (Centred)	−0.045	0.054	−0.840	0.401
Age 13 Teacher-Child Relationship (Centred)	0.036	0.034	1.042	0.297
Age 13 Corporal Punishment × Age 13 Teacher-Child Relationship (Product Term)	−0.030	0.063	−0.474	0.635
Age 11 Corporal Punishment (Centred)	0.096	0.049	1.954	0.051
Age 11 Corporal Punishment × Age 13 Teacher-Child Relationship (Product Term)	0.017	0.043	0.395	0.693
*Outcome Variable: Age 13 Aggression*				
Age 13 Teacher-Child Relationship (Centred)	−0.205	0.039	−5.219	<0.001
Age 13 Corporal Punishment (Centred)	0.138	0.047	2.914	0.004
Age 13 Corporal Punishment × Age 13 Teacher-Child Relationship (Product Term)	−0.113	0.059	−1.901	0.057
Age 11 Corporal Punishment (Centred)	−0.030	0.042	−0.721	0.471
Age 11 Teacher-Child Relationship (Centred)	0.013	0.042	0.319	0.750
Age 11 Corporal Punishment × Age 11 Teacher-Child Relationship (Product Term)	0.032	0.037	0.854	0.393
*Outcome Variable: Age 11 Aggression*				
Age 11 Teacher-Child Relationship (Centred)	−0.300	0.040	−7.439	<0.001
Age 11 Corporal Punishment (Centred)	0.230	0.056	4.103	<0.001
Age 11 Corporal Punishment × Age 11 Teacher-Child Relationship (Product Term)	−0.049	0.059	−0.838	0.402

*Est* parameter estimate, *S.E.* standard error

#### Females

**Fig. 6:**
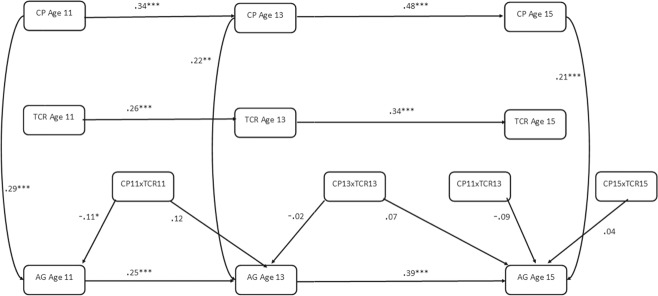
Autoregressive CLPM for Females when Testing Positive Teacher-Child Relationships as an Interactive Protective Factor. AG Aggression, CP Corporal Punishment, TCR Teacher-child Relationships, Lines represent regression paths. *p < 0.05, **p < 0.01, ***p < 0.001

**Fig. 7:**
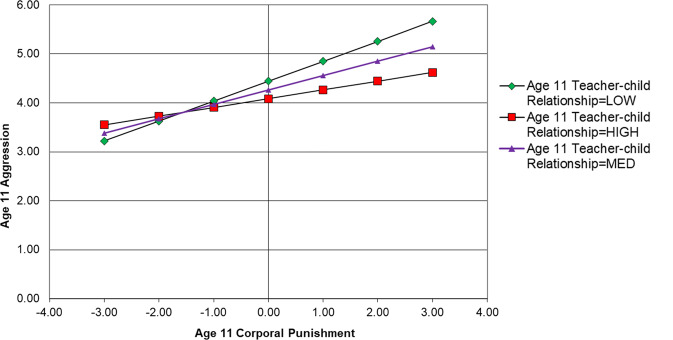
Simple Slopes Showing the Association Between Age 11 Corporal Punishment and Age 11 Aggression at High (+1 *SD*) Medium (centred mean) and Low (−1 *SD*) Levels of Positive Teacher-Child Relationship for Females

Positive teacher-child relationships had a significant protective main effect on concurrent aggression for females, across all age groups (age 11, β = −0.179, *p* > 0.05; age 13, β = −0.241, *p* > 0.05; age 15, β = −0.117, *p* > 0.05) (Table 4). The strongest main effect between positive teacher-child relationships and concurrent aggression for females was age 13, with the weakest being age 15. There was also a significant moderating effect of teacher-child relationships at age 11. Here it acted as an interactive protective factor in the relation between age 11 corporal punishment and age 11 aggression (β = −0.11, *p* < 0.05; Fig. 6). Simple slopes representing this interaction are displayed in Fig 7. There was no significant moderating effect of teacher-child relationships at age 13 nor 15. When considering lagged effects, there were no significant moderating effects of teacher-child relationships on the relation between corporal punishment exposure and later aggression.

**Table 4:** Path results for females of autoregressive CLPM testing teacher-child relationships as an interactive protective factor

Regression Paths	Estimate	*S.E*.	*Est./S.E*.	*Sig*.
*Outcome Variable: Age 15 Aggression*				
Age 15 Teacher-Child Relationship (Centred)	−0.117	0.039	−2.991	0.003
Age 15 Corporal Punishment (Centred)	0.211	0.061	3.489	<0.001
Age 15 Corporal Punishment × Age 15 Teacher-Child Relationship (Product Term)	0.043	0.059	0.735	0.463
Age 13 Corporal Punishment (Centred)	0.076	0.057	1.330	0.184
Age 13 Teacher-Child Relationship (Centred)	0.056	0.040	1.393	0.164
Age 13 Corporal Punishment × Age 13 Teacher-Child Relationship (Product Term)	0.073	0.064	1.138	0.255
Age 11 Corporal Punishment (Centred)	−0.138	0.049	−2.813	0.005
Age 11 Corporal Punishment × Age 13 Teacher-Child Relationship (Product Term)	−0.092	0.050	−1.860	0.063
*Outcome Variable: Age 13 Aggression*				
Age 13 Teacher-Child Relationship (Centred)	−0.241	0.047	−5.099	<0.001
Age 13 Corporal Punishment (Centred)	0.218	0.056	3.896	<0.001
Age 13 Corporal Punishment × Age 13 Teacher-Child Relationship (Product Term)	−0.024	0.050	−0.477	0.663
Age 11 Corporal Punishment (Centred)	0.017	0.059	0.286	0.775
Age 11 Teacher-Child Relationship (Centred)	−0.077	0.049	−1.559	0.119
Age 11 Corporal Punishment × Age 11 Teacher-Child Relationship (Product Term)	0.117	0.063	1.849	0.064
*Outcome Variable: Age 11 Aggression*				
Age 11 Teacher-Child Relationship (Centred)	−0.179	0.044	−4.079	<0.001
Age 11 Corporal Punishment (Centred)	0.293	0.054	5.386	<0.001
Age 11 Corporal Punishment × Age 11 Teacher-Child Relationship (Product Term)	−0.114	0.047	−2.444	0.015

In the present study, to ensure the robustness of results, sensitivity tests were conducted by repeating analyses using self-reported aggression, teacher-reported aggression and a combined child-teacher reported aggression measure. Results indicated that there was no substantive difference in the pattern of results based on informants of aggression.

## Discussion

The experience of corporal punishment is an important risk factor for later aggression; however, there is heterogeneity in the effects of corporal punishment and less is known about the factors that differentiate those exposed to harsh parenting who do versus do not subsequently show increases in their aggressive behaviour. Based on previous research suggesting that more positive teacher-child relationships can protect against subsequent behaviour problems, the goal of this original study was to explore whether positive teacher-child relationships also play a role in breaking the link between exposure to corporal punishment and the development of aggressive behaviours. Using a cross-lagged panel model with moderating effects in a large longitudinal sample of youth (aged 11,13 and 15), there was evidence that positive teacher-child relationships are protective but as a main effect and not an interactive effect. Indeed, the only significant interactive effects suggested that positive teacher-child relationships were more consistent with exacerbating the negative effects of corporal punishment; however, these effects were small and not consistent, i.e., limited to only a small number of the full set of comparisons conducted and differed based on the level of exposure to corporal punishment the child experienced.

This study sought to test the hypothesis that having a positive teacher-child relationship is a main effect and an interactive protective factor between corporal punishment and aggression. Consistent main effects were found for positive teacher-child relationships. For example, when considering adolescent aggression at age 11, age 13 and age 15, having a positive teacher child relationship was found to be a direct protective factor, suggesting that positive teacher-child relationships are beneficial in reducing aggression from early to middle adolescence. This was also the case when examining main effects stratified by gender, confirming that both males and females benefit from positive teacher-child relationships. More specifically, this study found that females were more likely to experience stronger positive relationships with their teachers at ages 11 and 13 when compared with males which is consistent with previous research (e.g., Birch & Ladd, 1997). However, when examining the main effects from the autoregressive cross-lagged panel model, the main effects between positive teacher-child relationships and concurrent aggression were stronger for males when compared to females. This suggests that for males, having a positive relationship with a teacher had a stronger direct protective effect against aggression than it did for females. These findings are consistent with previous research that has found that having a positive teacher-child relationship can act as a direct protective factor against developing problem behaviours (e.g. Pianta & Stuhlman, 2004; Silver et al., 2005; Vassallo et al., 2016). For example, positive teacher-child relationships protected against subsequent violence years later (Vassallo et al., 2016). Having a positive teacher-child relationship can result in developing positive affect and being able to have healthy interactions with peers (Hughes et al., 1999). However, still a majority of studies in this field have not used a longitudinal design or have not adjusted for previous levels of the constructs of interest. Further, few studies have focused on whether the effects of teacher-child relationships persist in adolescence as most previous research has focused on early childhood (De Laet et al., 2014). This study, which uses a cross-lagged panel design across three time points in adolescence, thus adds important evidence for the protective effects of teacher-child relationships beyond childhood.

The effect sizes were modest; however, this reflects the use of an autoregressive cross-lagged panel models to account for previous levels of the outcome variable (i.e., aggression). This can lead to a dramatic reduction in the association between the predictor and the outcome due to the partialling out of stability effects (Adachi & Willoughby, 2015). However, studies have found that although smaller main effects might be found using this statistical approach, those small effects can still be meaningful and important, especially when they accumulate over time (Adachi & Willoughby, 2015). The strength of the autoregressive cross-lagged panel model is that it allows the user to ensure that any cross-lagged effects did not simply reflect the association between those two variables at the previous time point.

In contrast to the main effects of teacher-child relationships, the evidence for a moderating effect of teacher-child relationships on concurrent and later aggression was much less consistent. While there were some indications of possible moderation that merit further exploration in future research, the significance and direction of effects were inconsistent across gender and developmental stage. Further, the significant effects that did emerge tended to relate to concurrent effects which have an ambiguous interpretation given that the constructs involved are not temporally ordered. Future research could, therefore, examine the moderating impact of teacher-child relationships over shorter timescales to provide further illumination on this issue.

When examining the interactive protective effect of positive teacher-child relationships for males and females separately, this study found that having a positive teacher-child relationship at age 11 was an interactive protective factor for females, but not for males. For 11-year-old females, having a stronger relationship with a teacher resulted in a stronger interactive protective effect against corporal punishment. Gender differences were also found when examining the protective effect of positive teacher-child relationships at age 15. For 15-year-old males, having a positive teacher-child relationship was found to have a significant interaction between corporal punishment at age 15 and concurrent aggression. The direction of the interaction suggests that at this age, having a positive teacher-child relationship did not protect against the adverse effect of corporal punishment, but instead, exacerbated it. Previous research has found that the protective effect of a variable can differ depending on the degree of risk the young person is exposed to (e.g., Dubow et al., 2016). This is evident when examining this significant interaction for males. For example, the results show that for 15-year-old males, having a positive relationship with a teacher is a protective factor for those who have been exposed to low levels of corporal punishment. However, when a 15-year-old male is exposed to higher levels of corporal punishment, the protective capability of a positive teacher-child relationship is reduced, and levels of aggression are similar to those who have a poor relationship with their teachers. Future research should consider the examination of gender differences in relation to the degree of exposure to corporal punishment and its impact on subsequent aggression.

Taken together, the results of this study have potential implications for aggression prevention programmes. Specifically, they suggest that efforts to strengthen teacher-child relationships remain important in the adolescent period. Violence prevention efforts often focus on the individual and their skills (e.g., social skills, self-control) (Farrington et al., 2016), or the family environment and even school-based interventions primarily focus on child competencies; however, these findings suggest that teacher-child relationships are a potentially promising target for intervention that can complement existing targets.

When discussing some of the limitations of the currently study, it is important to note that capturing the extent of corporal punishment is often difficult due to it going either unreported or unrecognised by both parents and children (Fréchette et al., 2015; Straus & Stewart, 1999; Straus, 2010). It may also be the case that disclosures by children who experience corporal punishment by a family member may not disclose their experiences to others because they do not want to appear to be a troublemaker or a liar (Krahé, 2001). Recall accuracy could influence self-reported experiences of corporal punishment as well as the fact that it is a controversial form of discipline which is sometimes believed to be an appropriate punishment (Fréchette et al., 2015). In Zurich, where data for the current study was collected, corporal punishment is lawful in the home under the parents “right of correction”. It could also be difficult to differentiate between physical abuse and corporal punishment due to the potential overlap of their definitions. Although data for this study was extracted from a broader study that incorporated parenting interventions, these intervention conditions were randomly assigned and overall, there was little evidence that the parenting intervention had a substantial or lasting effect. Methodologically, while formal comparison tests such as the Satorra-Bentler test were outside the scope of the current study, future studies would benefit from replicating the models used in this study and conducting formal model comparison. Furthermore, it is important to consider potential sample bias towards lower levels of experienced corporal punishment due to it being parents and caregivers giving consent to participate in the study. It is also worth noting the possibility that those who had worse relationships with teachers and high levels of aggression might be more likely to drop out of school, which might have the effect of attenuating their relationships. There is also the possibility that students might under-report poor relationships with teachers because of the school setting of data collection. Future studies would benefit from exploring the effects of confounds not included in the scope of this study such as peer relationships, academic achievement, and socioeconomic factors.

## Conclusion

The link between corporal punishment and adolescent aggression has been well established; however, little is known about the protective capability of positive teacher-child relationships across different developmental stages within adolescence. Using a longitudinal study design that employed an autoregressive cross-lagged panel model, the results of the current study recommended promoting interventions that seek to improve teacher-child relationships in the adolescent period. Having a positive teacher-child relationship was found to reduce levels of adolescent aggression across all age groups and in both males and females. Generally, there was little evidence that this effect was moderated by an adolescent’s exposure to corporal punishment, suggesting that such interventions would be suitable for youth with varying levels of exposure to this risk factor. Future research should explore possible indications of moderation identified in this study, particularly in longitudinal studies with shorter time lags that can capture the more proximal effects of corporal punishment and teacher-child relationships on aggression.

## Supplementary information

Supplementary Information
