# Supplementary material for: Disrupting the Link between Corporal Punishment Exposure and Adolescent Aggression: The Role of Teacher-Child Relationships
Source: J Youth Adolesc. 2022 Sep 13;51(12):2265–80. doi: 10.1007/s10964-022-01666-6 (PMC9596518; doi:10.1007/s10964-022-01666-6)
Supplement: Supplementary file 1 — Supplementary Information [file 10964_2022_1666_MOESM1_ESM.docx]

**Supplementary Materials**

| **S1** Correlations between Corporal Punishment, Teacher-Child Relationship and Aggression | | | | | | | | | |
| --- | --- | --- | --- | --- | --- | --- | --- | --- | --- |
|  | 1 | 2 | 3 | 4 | 5 | 6 | 7 | 8 | 9 |
| Corporal Punishment | | | | | | |  |  |  |
| 1. Age 11 |  | .33** | .25** | -.15** | -.10** | -.04 | .32** | .17** | .17** |
| 2. Age 13 |  |  | .41** | -.12** | -.17** | .02 | .12** | .28** | .21** |
| 3. Age 15 |  |  |  | -.01 | -.10 | -.12** | .09** | .16** | .28** |
| Teacher-Child Relationship | |  |  |  |  |  |  |  |  |
| 4. Age 11 |  |  |  |  | .25** | .15** | -.32** | -.22** | -.12** |
| 5. Age 13 |  |  |  |  |  | .32** | -.22** | -.32** | -.19** |
| 6. Age 15 |  |  |  |  |  |  | -.16** | -.18** | -.24** |
| Aggression | |  |  |  |  |  |  |  |  |
| 7. Age 11 |  |  |  |  |  |  |  | .43** | .34** |
| 8. Age 13 |  |  |  |  |  |  |  |  | .55** |
| 9. Age 15 |  |  |  |  |  |  |  |  |  |
| **p<.05* ** *p*<.01. | | | | | | |  |  |  |
